# A case of systemic lupus erythematosus presenting as bilateral avascular necrosis of femur

**DOI:** 10.1186/s13104-016-2198-9

**Published:** 2016-08-05

**Authors:** Madura Adikari, Aloka Gunawardane, Sachithra Illangantilaka, Himantha Atukorale, Jeevanie Rubasinghe

**Affiliations:** Rheumatology Unit (Special), National Hospital of Sri Lanka, Colombo, Sri Lanka

## Abstract

**Background:**

Avascular necrosis occur as a result of diverse etiology. Chronic inflammatory conditions such as systemic lupus erythematosus considered as a recognize cause. Many cases were reported in systemic lupus erythematosus after treating with corticosteroids. We report a case of a corticosteroid naïve patient presented as bilateral avascular necrosis of femoral head and later progressed to a case of systemic lupus erythematosus.

**Case presentation:**

A 26 year old lady presented with right sided hip pain and diagnosed as avascular necrosis of the femoral head. After 6 months she presented a similar pain in left hip, which revealed avascular necrosis of left femoral head as well. A probable cause for her clinical presentation could not be found after extensive clinical and laboratory evaluation. Patient reported high erythrocyte sedimentation rate persistently, and over the next few years progressed as a case of systemic lupus erythematosus.

**Conclusion:**

Above case illustrated avascular necrosis could be an early musculoskeletal manifestation of systemic lupus erythematosus even in the absence of corticosteroid administration.

## Background

Avascular necrosis (AVN) also known as osteonecrosis occur as a result of diverse etiology. Chronic inflammatory disorders, high-dose corticosteroids, excess alcohol intake, smoking, trauma, sickle cell disease, infections such as human immunodeficiency virus (HIV), tuberculosis, meningococcal infections are some of the common causes AVN of bone [1, 2]. Systemic lupus erythematosus (SLE) is an autoimmune, chronic inflammatory multisystem connective tissue disease and AVN of bone is a well recognized complication of SLE [3]. Musculoskeletal manifestation of SLE is diverse including arthralgia, sinovitis, myositis, myopathy and AVN of bone which occur in variable frequencies at various stages of the disease. SLE can evolve and progress over years with various multisystem clinical manifestations before definitive diagnosis made and a definitive diagnosis can be delayed up to 5 years [a]. Many cases were reported of multiple AVN of bones in patient with SLE, who has been treated with corticosteroids. AVN in single or multiple joints as an early manifestation of SLE in a patient who has not previously treated with corticosteroids is not a known association [3–5]. We report a case of a steroid naïve patient presented as bilateral AVN of femoral head and later progressed to SLE.

## Case report

A 26 year old female has been referred to our clinic with right sided hip pain of sub acute onset. Her pain worsened with movement and weight bearing. On clinical examination, she had restricted range of movement with pain and mild joint tenderness. On plain x-ray she found to have AVN of right femoral head suggested by classical crescent sign with preserved articular margins on plain film and underwent extensive evaluation for a cause. Apart from malaise, fatiguability and moderately elevated erythrocyte sedimentation rate (ESR) of 65 mm in 1st h no other symptom or laboratory feature could be found. Her hemoglobin level was 10.6 g/dL and white cell count, platelet count, C-reactive protein was normal. After 6 months she presented with a similar pain in left hip and subsequent work up revealed AVN of left femoral head as well on plain x-ray and findings were similar to the previous right sided hip joint. Other than above presentation she had no other complains at that point. Her past medical history was not significant, she gave birth to a healthy term baby 7 months prior to above presentations and her obstetric history was uneventful. She did not consume alcohol and was a non-smoker. She was not on any form of corticosteroid or other medications prior to the event except nutritional supplements during antenatal period and she has not taken any indigenous medicines. There was no family history of connective tissue or other autoimmune condition of note. At the time she of the second event of hip pain she underwent a comprehensive work up with involvement of a multidisciplinary specialists. In view of persistently high ESR and multiple AVN of bones, she underwent a work up for chronic infections such as tuberculosis, and for autoimmune connective tissue diseases with serological studies including antinuclear antibody (ANA), Anti Smith antibody (Sm), serum complements C3 and C4, Anti Ro and Anti La antibodies. She was also screen for Human Immunodeficiency Virus (HIV) infection, Hepatitis B and C infections. Hematological evaluation for sickle cell disease, thrombophilic conditions including anti-cardiolipin antibodies IgG and lupus anticoagulant test, beta-2 glycoprotein 1 both IgM and IgG. All of the above tests were negative. A probable cause could not be ascertained despite above extensive workup. She has been continuously followed up with a future plan of hip joint replacement. During the subsequent period patient revealed that she felt mild fatiguability and hair loss continuously, which she considered not significant and did not report to the doctor hence further workup was not done for next 4 years. She did not take any medications except simple paracetamol for hip pains. After about 4 years of above events she reported significant hair loss, which was unusually high according to her. On further assessment she found to have isolated thrombocytopenia of less than 100,000/mm^3^ on repeated blood counts and also continuously high ESR, at times exceeding 100 mm/1st h. Patient was re-evaluated considering above developments. She had positive ANA with titre above 1:160 and a positive anti double stranded DNA (dsDNA) antibody with titre of 233 IU/mL. Both these tests were negative 3 years back. She did not report abnormality in urinalysis or neuropsychiatric manifestations of SLE. In light of above clinical manifestations and serological studies, definite diagnosis of SLE was made according to SLICC 2012 classification criteria. Her coagulation screening was repeated and was negative although beta-2 glycoprotein 1 was not repeated. Her initial presentation of bilateral AVN of femoral head 4 years back was ascertained as related to evolving autoimmune disease, since no other etiology was evidenced. Patient was prescribed hydroxy-chloroquine and simple analgesics in her subsequent management and total hip joints replacement surgery was planned.

## Discussion

AVN or osteonecrosis is caused by loss of blood supply to a part of bone leading to bone necrosis and collapse [1]. Diverse etiologies have been described for AVN. Chronic inflammatory conditions such as SLE considered as a well known cause [2]. In a patient of SLE, several factors can lead to bone ischemia and AVN include Raynaud’s phenomenon, vasculitis, fat emboli, corticosteroids, and the antiphospholipid syndrome [3, 6]. Although the above patient had evidence of a chronic inflammatory disorder such as malaise and high ESR at the outset, she didn’t have sufficient criteria fulfilling the diagnosis of SLE or any other autoimmune disorder and she was not on any form of corticosteroid treatment. AVN is known to associated with pregnancy although in this patients onset was well beyond the post partum period of 7 and 13 months. Many reviews observed that AVN often develops shortly after the onset of high-dose corticosteroid therapy [7]. Numerous case reports and case series were reported AVN in already diagnosed SLE patients and after initiation of corticosteroid therapy, some of them reported AVN in multiple sites [6].

Gontero et al. [3] conducted a study among 158 SLE patients and 15 patients (9.5 %) had AVN, however all were on corticosteroids for variable durations and dosages, but no association was found with the dosage or disease activity of SLE with the development of AVN. Another study conducted to evaluate the prevalence of AVN in patient with SLE and antiphospholipid syndrome (APLS) and who were not on corticosteroids, using magnetic resonant imaging (MRI) [5]. This study evaluated asymptomatic patients including 19 SLE patients, 30 primary APLS patients and 30 healthy subjects, all of them were not on corticosteroids. AVN was not reported in any of the above SLE patient or healthy subjects although six APLS patients revealed AVN on MRI studies [5]. However our patient was repeatedly negative for APLS screening. In our rigorous literature search we could not find a reporting of AVN either single or multiple sites as early manifestation of steroid naïve SLE patient. Above case illustrates a patient with a features chronic inflammatory disorder developing bilateral AVN in femoral heads and later progressed to SLE over next few years. Since we couldn’t find an alternative explanation of AVN in this patient and presence of clinical and laboratory features of chronic inflammatory disorder justified considering all above manifestations were related to an evolving case of SLE, which she ultimately met the diagnostic criteria.
